# Embryonic Morphogen Nodal Promotes Breast Cancer Growth and Progression

**DOI:** 10.1371/journal.pone.0048237

**Published:** 2012-11-07

**Authors:** Daniela F. Quail, Guihua Zhang, Logan A. Walsh, Gabrielle M. Siegers, Dylan Z. Dieters-Castator, Scott D. Findlay, Heather Broughton, David M. Putman, David A. Hess, Lynne-Marie Postovit

**Affiliations:** 1 Department of Anatomy & Cell Biology, University of Western Ontario, London, Ontario, Canada; 2 Human Oncology and Pathogenesis Program, Memorial Sloan Kettering Cancer Center, New York, New York, United States of America; 3 Department of Physiology and Pharmacology and Robarts Research Institute, London, Ontario, Canada; University of Tampere, Finland

## Abstract

Breast cancers expressing human embryonic stem cell (hESC)-associated genes are more likely to progress than well-differentiated cancers and are thus associated with poor patient prognosis. Elevated proliferation and evasion of growth control are similarly associated with disease progression, and are classical hallmarks of cancer. In the current study we demonstrate that the hESC-associated factor Nodal promotes breast cancer growth. Specifically, we show that Nodal is elevated in aggressive MDA-MB-231, MDA-MB-468 and Hs578t human breast cancer cell lines, compared to poorly aggressive MCF-7 and T47D breast cancer cell lines. Nodal knockdown in aggressive breast cancer cells via shRNA reduces tumour incidence and significantly blunts tumour growth at primary sites. *In vitro*, using Trypan Blue exclusion assays, Western blot analysis of phosphorylated histone H3 and cleaved caspase-9, and real time RT-PCR analysis of *BAX* and *BCL2* gene expression, we demonstrate that Nodal promotes expansion of breast cancer cells, likely via a combinatorial mechanism involving increased proliferation and decreased apopotosis. In an experimental model of metastasis using beta-glucuronidase (GUSB)-deficient NOD/SCID/mucopolysaccharidosis type VII (MPSVII) mice, we show that although Nodal is not required for the formation of small (<100 cells) micrometastases at secondary sites, it supports an elevated proliferation:apoptosis ratio (Ki67:TUNEL) in micrometastatic lesions. Indeed, at longer time points (8 weeks), we determined that Nodal is necessary for the subsequent development of macrometastatic lesions. Our findings demonstrate that Nodal supports tumour growth at primary and secondary sites by increasing the ratio of proliferation:apoptosis in breast cancer cells. As Nodal expression is relatively limited to embryonic systems and cancer, this study establishes Nodal as a potential tumour-specific target for the treatment of breast cancer.

## Introduction

Two classical and fundamental hallmarks of cancer include enhanced proliferation and evasion of apoptotic signals [1], [2]. Normally, epithelial cells require signals from their microenvironment to trigger entrance into a proliferative state. In contrast, cancer cells exhibit a reduced dependence on mitogenic factors from their microenvironment, and can enter a proliferative state in response to their own deregulated growth signals. In breast cancer, patients bearing tumours that express high levels of the proliferation marker nuclear antigen Ki67, concomitant with mutations that mitigate apoptotic programmes, exhibit accelerated disease progression and poor prognosis [3]–[6]. Elucidating factors that regulate proliferative programmes and that, therefore, cause susceptibility to tumour cell expansion is of interest in order to develop effective targeted cancer therapies.

In addition to enhanced proliferation and evasion of apoptosis during cancer progression, aberrant expression of stem cell factors within breast tumours sustains aggressive phenotypes, and is associated with growth-promoting profiles in tumour cells and their microenvironments. One example of a stem cell factor that is associated with cancer progression is Nodal, an embryonic morphogen and member of the Transforming Growth Factor-Beta (TGF-β) superfamily. Nodal expression is limited to pluripotent stem cells during embryonic development and to specialized dynamic adult tissue (such as the cycling endometrium), but is re-expressed to induce growth programmes in cancers such as melanoma, prostate cancer, endometrial cancer, glioma, pancreatic cancer and hepatocellular carcinoma [7]–[15]. In accordance with its documented contribution to tumour growth, Nodal has recently been linked to proliferation in a variety of normal physiological systems. For example, Harrison and colleagues have studied Nodal signalling in human endometrium during the various phases of remodelling, and found that Nodal is highly expressed throughout the proliferative and early secretory phases, and is abruptly downregulated by the mid-secretory phase [13]. In addition, Salomon and colleagues have found that Nodal and members of the Nodal signalling pathway are cyclically expressed during mammary gland remodelling. In particular, Nodal, Cripto, ALK4, and SMAD4 are upregulated during lactational expansion of alveolar epithelial tissue, and downregulated during involution (marked by widespread apoptosis) in BalbC mice [16], [17]. Together, these studies suggest that Nodal may play a role in promoting proliferative phenotypes in dynamic epithelial cell types.

A recent study reported that Nodal is positively correlated with disease progression in breast cancer patients, such that it is expressed to a higher level in poorly differentiated, invasive lesions as compared to benign and early stage disease [18]. This study further demonstrated that inhibition of Nodal signalling in aggressive breast cancer cell lines reduces proliferation and induces apoptosis *in vitro* [18]. In addition, transient inhibition of Nodal in MDA-MB-231 breast cancer cells with Morpholino oligonucleotides has been shown to delay tumourigenesis in nude mice, concomitant with reduced proliferation (by Ki67 staining) and elevated apoptosis (by TUNEL staining) [19]. It is not known, however, if these trends extend to gain-of-function models or if Nodal supports breast cancer growth at both primary and secondary sites.

Here, we demonstrate that stable Nodal knockdown significantly blunts tumour growth in an orthotopic mouse model of tumourigenesis. *In vitro,* we confirmed that Nodal inhibition in aggressive breast cancer cell lines decreases proliferation whilst inducing apoptosis, and further demonstrated that inhibition of the type 1 receptor (ALK4/7) similarly affects breast cancer proliferation. We also determined that Nodal over-expression increases proliferation and decreases apoptosis in T47D cells which do not normally express high levels of this protein. Lastly, using a unique experimental metastasis assay, we found that although Nodal does not affect the *number* of micrometastases in the lung (i.e. seeding efficiency), it enhances proliferation:apoptosis ratios in micrometastases in favour of tumourigenic growth. Indeed, these experiments indicated that Nodal is required for growth progression to macrometastases at secondary sites. Our findings demonstrate that Nodal mediates growth of breast cancer cell lines *in vitro* and at multiple sites *in vivo*.

## Methods

### Cell Lines and Treatments

Two well-differentiated, breast cancer cell lines (MCF-7 and T47D) and three poorly-differentiated, cell lines (MDA-MB-231, MDA-MB-468 and Hs578t) [20] were used. All cancer cell lines were obtained from the American Type Culture Collection (ATCC) and were maintained as per instructions. The phenotypes of these cells were verified by ATCC in accordance with protocols available on the ATCC website. To increase Nodal signalling, we used a Nodal expression vector (versus an empty pcDNA3.3 vector; pcDNA™3.3-TOPO® cloning kit; Invitrogen) as previously described [21]. We also employed recombinant human Nodal (rhNodal; R&D). To decrease Nodal signalling, we used Nodal-targeted shRNAs (versus scrambled control shRNAs) as previously described [21]. Two Nodal-targeted shRNAs were used, a HuSH-29mer (Id: GI311711; Origene) and a GIPZ lentiviral shRNAmir (Id: V2LHS_155453; Open Biosystems) to rule-out off-target effects. Transfection was performed with Arrest-In (Open Biosystems) or Lipofectamine (Invitrogen) as per manufacturer instructions. For stable selection, Puromycin (200–450 ng/mL) or Geneticin (G418; 800 ng/mL) was used. To inhibit Nodal signalling, we also used SB431542. SB431542 selectively inhibits Activin, TGF-β and Nodal signalling but not BMP signalling. In addition, SB431542 does not affect components of the ERK, JNK, or p38 MAP kinase pathways [22].

### RNA Extraction and RT-PCR

RNA isolation was performed using the Perfect Pure RNA cultured cell kit (5 Prime), and DNAse was used to degrade genomic DNA. Reverse transcription was performed using 2 µg of RNA and a High Capacity cDNA Reverse Transcription kit (Applied Biosystems). Real-time PCR was performed with TaqMan® gene expression human primer/probe sets. For a list of primer/probes, see **Table S1**. For analysis of *BAX* and *BCL2* gene expression in response to treatments, Ct values were normalized to *HPRT1*, and compared using the ΔΔCt method. Variability in housekeeping gene expression across cell lines confounded results obtained with real time-RT-PCR. Hence, semi-quantitative RT-PCR was used to measure Nodal receptor components across breast cancer cell lines using H9 hESC mRNA as a positive control. For semiquantitative PCR, 1/20^th^ of the cDNA reaction was used as template for amplification using AmpliTaq Gold® 360 Master Mix (Applied Biosystems). Validated primer probes were used as described above. The loading control *HPRT1* was amplified with forward primer: 5′-atggcgacccgcagccctgg-3′, and reverse primer: 5′-ctggcgatgtcaataggactccagatgtttcc-3′. The following cycling conditions were used: 95° for 30 sec, 55° (*ALK4, ALK7, Cripto*) or 64° (*HPRT1*) for 30 sec, and 72° for 1 min. ALK4 and HPRT1 were subjected to 30 cycles. ALK7 and Cripto were subjected to 35 cycles.

### Western Blotting

Protein lysates were prepared using Mammalian Protein Extraction Reagent (M-PER; Thermo Scientific) and Halt Protease Inhibitor Cocktail (Thermo Scientific) as per manufacturer’s instructions. Equal amounts of protein were reduced and separated by SDS-polyacrylamide gel electrophoresis, and transferred onto Immobilon-P membranes (Millipore). Membranes were blocked in 5% milk, incubated with primary antibody, washed, and incubated with horseradish peroxidase-labelled secondary antibody. For a list of primary antibodies see **Table S2**. Secondary antibodies were detected by enhanced chemiluminescence (Super Signal; Pierce). In accordance with previous studies [9], [11], [23], [24], three banding locations were detected for Nodal: Pro-Nodal at ∼39 kDa, pre-pro-Nodal at ∼50 kDa, and mature Nodal at ∼15 kDa. The 50 kDa species is highly variable due to differences in post-translational modifications and protein lysate handling, and the 15 kDa band is poorly abundant in protein lysate. For consistency, we used the 39 kDa band to assess Nodal expression in lysates and the 15 kDa band in conditioned medium, as previously described [21], [24].

### Tumour Assays in Nude Mice

All experiments involving animals were approved by the Animal Use Subcommittee at the University of Western Ontario.

#### Nodal loss-of-function flank tumour assay

MDA-MB-468 cells were transfected with a Control HuSH shRNA, or a Nodal-targeted HuSH shRNA. 2,500,000 cells in 100 µL of RPMI+Matrigel (1∶1) were injected into the right flank of 6–8 week old athymic Nude-Foxn1^nu^ mice. Tumour measurements were taken twice per week and a digital caliper was used to measure Length × Width × Depth of the tumour upon excision in order to calculate volume.

#### Nodal loss-of-function orthotopic tumour assay

MDA-MB-231 cells were transfected with a Control GIPZ shRNA, or a Nodal-targeted GIPZ shRNA. 500,000 cells in 50 µL of RPMI were injected into the mammary fat pad via the nipples of 6–8 week old athymic Nude-Foxn1^nu^ mice. Tumour measurements were taken as described above.

### *In vitro* Cell Growth Curves

Cells were seeded into 6-well plates (100,000 cells/well) and counted over 4 days. Media containing dead and alive cells was collected. Attached cells were harvested using Trypsin, combined with media, spun down, and resuspended with Trypan Blue. A Countess automated cell counter (Invitrogen) was used to calculate total cell number, live cells, dead cells, and viability.

### Cell Trace Violet Proliferation Assays

Cells were starved 21–22 hours and then labelled with 2.5 µM (T47D lines) or 5 µM (MDA-MB-231 lines) Cell Trace Violet (CTV, Invitrogen) as per the manufacturer’s instructions. Briefly, culture medium was removed from cells and replaced with CTV diluted to 2.5 or 5 µM in pre-warmed phosphate-buffered saline (PBS). Cells were incubated 20 min at 37°C after which CTV solution was removed and cells were washed twice with pre-warmed complete medium and then left in fresh medium for 4–6 days. Cells were harvested via trypsinization and then washed in FACS buffer (PBS+1% FBS+2 mM EDTA) before flow cytometric acquisition. Fluorescence-activated cell sorting (FACS) was performed on an LSRII (Becton Dickinson, Mississauga ON) calibrated with CaliBRITE Beads (Becton Dickenson, Mississauga ON). Live cell singlets were gated based on forward and side-scatter properties. Analysis was performed using FlowJo© software (Tree Star, Ashland, OR, USA, Version 9.5.2).

### TUNEL Staining

Cells were grown on glass coverslips to ∼50% confluence and then fixed with 4% paraformaldehyde. The DeadEnd colorimetric TUNEL system (Promega) was used to measure apoptosis as per instructions. The % TUNEL positive cells were determined by counting the number of positive cells in 3 fields of view on each slide taken at 40X. This number was divided by the total number of cells in these fields. At least 4 slides were used per experimental group.

### Immuonofluorescence

Cells were fixed with 4% paraformaldehyde, made permeable with 20 mM Hepes, 0.5% TritonX-100 and blocked with serum-free protein block (DAKO). Primary antibodies were diluted in antibody dilutent (DAKO) to the concentrations outlined in **Table S2**, and appropriate fluorochrome-conjugated secondary antibodies were used according to manufacturer recommendations. Nuclei were stained with DAPI (0.1 mg/mL; Invitrogen/Molecular Probes, Eugene, OR), and images were obtained using confocal microscopy (Zeiss 510 META, Carl Zeiss Inc.).

### Experimental Metastasis Assay in NOD/SCID/MPSVII Mice

500,000 cells in 700 µL Ca^2+^-free HBSS were injected into the tail vein of NOD/SCID/MPSVII mice. Mice were sacrificed at 4 weeks (to assess micrometastases) and 8 weeks (to assess macrometastases). Lung, brain, and liver from transplanted NOD/SCID/MPSVII mice were frozen in OCT embedding medium (Sakura Finetek, Torrance, CA) for histochemical analysis. Serial sections at 10-µm thickness, were fixed in 10% buffered formalin (Sigma-Aldrich, St. Louis, MO), and blocked with mouse-on-mouse reagent (Vector Laboratories, Burlingame, CA). Sections were analyzed for human cells by colourimetric detection of ubiquitous GUSB activity in human cells as previously described using napthol AS-BI β-D-glucuronide (Sigma-Aldrich) substrate [25], and counterstained with haematoxylin. Metastases that were<100 cells were considered ‘micro’, while metastases that were>100 cells were considered ‘macro’. For each mouse organ, 3–6 sections were acquired from evenly spaced areas through the tissue, and the average number of metastases per mouse organ was calculated. The proliferation-to-apoptosis ratio was determined by counting Ki67 and TUNEL positive nuclei in matched serial sections. Briefly, tissues were formalin-fixed and paraffin-embedded and immunohistochemical staining on this tissue was conducted using a human-specific Ki67 antibody (**Table S2**) as per manufacturer suggestion. The DeadEnd colorimetric TUNEL system (Promega) was used to measure apoptosis as per instructions. The proliferation-to-apoptosis ratio was determined by counting Ki67 and TUNEL positive nuclei in matched serial sections. At least 3 pairs of serial sections, evenly spaced through the tissue, were averaged per mouse to yield an average proliferation-to-apoptosis score for that animal.

### Statistical Analyses

Statistics were performed using SigmaStat (Dundas Software), and validated through the biostatistical support unit at the University of Western Ontario. All parametric data was analysed using a one-way ANOVA and a Tukey–Kramer Comparisons Post-Hoc test. All non-parametric data was analyzed using an ANOVA on Ranks followed by the Mann-Whitney rank-sum test, and expressed as median ± interquartile range. A student’s t-test was used to compare two items. All statistical tests were two-sided, and data were considered statistically significant at *p<*0.05.

## Results and Discussion

### Expression of Nodal, ALK4, ALK7 and Cripto in Breast Cancer Cell Lines

**Figure 1:**
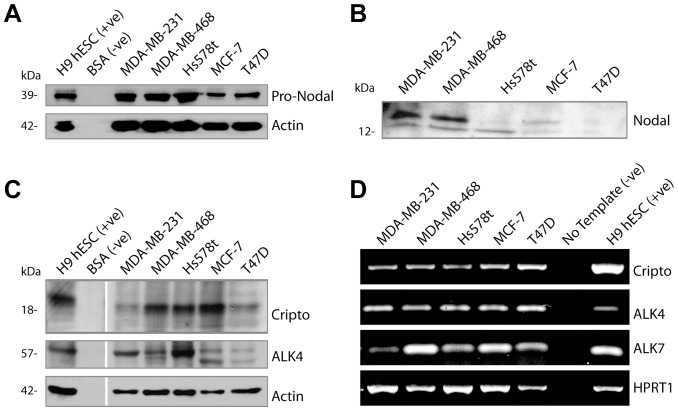
Nodal and its receptors are expressed by breast cancer cells. (A) Western blot analysis of Nodal in poorly differentiated (MDA-MB-231, MDA-MB-468, Hs578t) and well differentiated (MCF-7, T47D) breast cancer cells. Nodal expression is elevated in highly aggressive MDA-MB-231, MDA-MB-468 and Hs578t breast cancer cells compared to poorly aggressive MCF-7 and T47D breast cancer cells. H9 human embryonic stem cells were used as a positive control. ∼39 kDa Pro-Nodal band is depicted and Actin is used as a loading control. (B) Western blot analysis of Nodal in the conditioned medium of MDA-MB-231, MDA-MB-468, Hs578t, MCF-7 and T47D breast cancer cells. Nodal secretion is elevated in MDA-MB-231 and MDA-MB-468 breast cancer cells compared to Hs578t, MCF-7 and T47D breast cancer cells. H9 human embryonic stem cells were used as a positive control. ∼15 kDa secreted Nodal band is depicted. (C) Western blot analyses of Cripto and ALK4 in MDA-MB-231, MDA-MB-468, Hs578t, MCF-7 and T47D breast cancer cells. All cell lines make these members of the Nodal receptor complex. H9 human embryonic stem cells were used as a positive control. Actin is used as a loading control. (D) RT-PCR analysis of *Cripto*, *ALK4* and *ALK7* mRNA expression levels in MDA-MB-231, MDA-MB-468, Hs578t, MCF-7 and T47D breast cancer cells. All cell lines transcribe these members of the Nodal receptor complex. H9 human embryonic stem cells were used as a positive control. *HPRT1* is used as a loading control.

Through Western blot analyses, we determined that Nodal protein is elevated in cell lysates and in conditioned media from poorly-differentiated Hs578t, MDA-MB-231 and MDA-MB-468 breast cancer cell lines compared to well-differentiated MCF-7 and T47D cell lines (**Fig. 1A,B**). This is consistent with previous reports that show high Nodal expression in aggressive melanoma, prostate, and breast cancer cell lines compared to poorly aggressive lines [7], [9], [11], [18].

Nodal signals through interactions with Cripto-1 and the Activin-Like Kinase type I (ALK4/7) and type II (ActRIIB) receptor complex. Activation of this receptor complex leads to SMAD2/3 phosphorylation, and subsequent induction of Nodal-dependent gene expression [26]. It has been reported that Nodal receptor components are expressed at varying levels in prostate cancer cell lines [27]. In order to ensure the breast cancer cell lines used in this study have the potential to respond to alterations in Nodal, we measured the expression of the members of the Nodal receptor complex. Accordingly, we determined that ALK and Cripto protein are present in Hs578t, MDA-MB-231, MDA-MB-468, T47D and MCF-7 breast cancer cell lines at varying levels and that all of these cell lines express *ALK4*, *ALK7* and *Cripto* mRNA (**Fig 1C,D**). This suggests that these cell lines are able to respond to and carry out Nodal-induced signal transduction. Since they secrete higher levels of Nodal, we decided to use MDA-MB-468 and MDA-MB-231 cells in our loss-of-function models; and because they secrete very low levels of Nodal, but still express Nodal receptors, we chose to use T47D cells to study how the up-regulation of Nodal expression affects breast cancer cells.

### Nodal Knockdown Prevents Tumourigenesis using Aggressive Breast Cancer Cells

**Figure 2:**
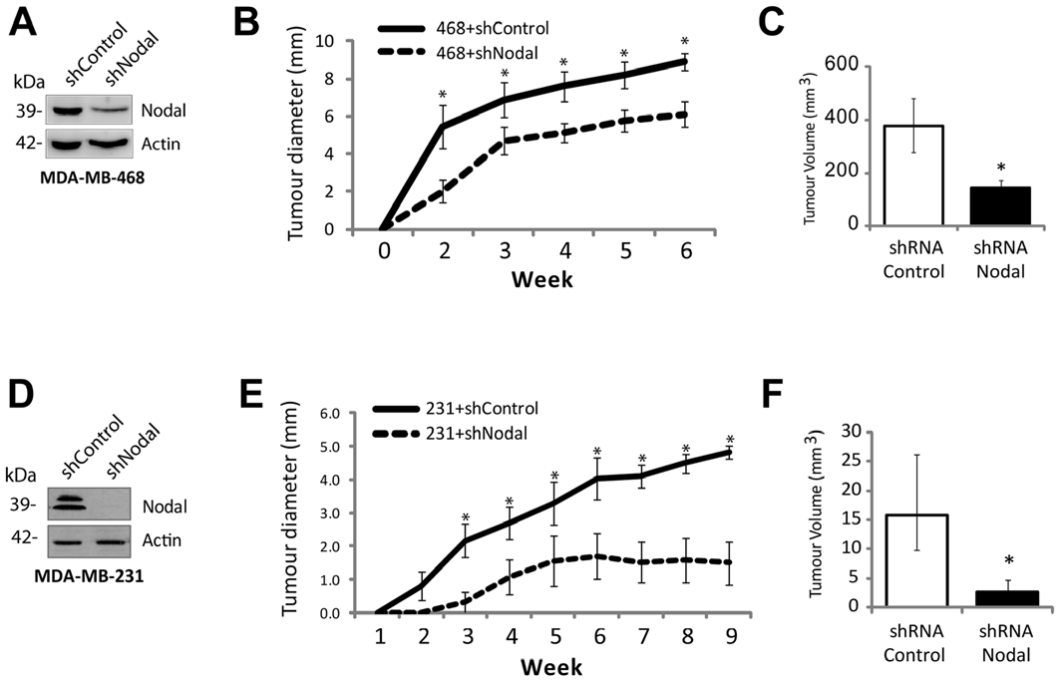
Nodal knockdown reduces breast cancer tumour growth *in vivo*. (A) Western blot confirming Nodal knockdown by shRNA in MDA-MB-468 cells. The ∼39 kDa Pro-Nodal band is presented and Actin is used as a loading control. (B) 2.5 million MDA-MB-468 cells transfected with Control shRNA (468+shControl) or a Nodal-targeted shRNA (468+shNodal) were injected with Matrigel into the flanks of nude mice, and tumour diameter was measured over the course of 6 weeks. 468+shControl cells formed significantly larger tumours compared to 468+shNodal cells (n = 8, p<0.05). Values represent mean tumour diameter (mm) ± standard error of the mean (S.E.M.). (C) Tumour volume of MDA-MB-468-derived tumours excised after 6 weeks. Bars represent mean tumour volume (mm^3^) ± S.E.M. (D) Western blot confirming Nodal knockdown by shRNA in MDA-MB-231 cells. The ∼39 kDa Pro-Nodal band is presented and Actin is used as a loading control. (E) 0.5 million MDA-MB-231 cells transfected with Control shRNA (231+shControl) or Nodal-targeted shRNA (231+shNodal) were orthotopically injected through the nipple into the mammary fat pads of nude mice, and tumour diameter was measured over the course of 9 weeks. 231+shControl cells formed significantly larger tumours compared to 231+shNodal cells (n = 10, p<0.05). Values represent mean tumour diameter (mm) ± S.E.M. (F) Tumour volume of MDA-MB-231-derived tumours excised after 9 weeks. Bars represent mean tumour volume (mm^3^) ± S.E.M.

Given that Nodal is associated with aggressive cancers and breast cancer cell lines, we first sought to determine whether stable Nodal knockdown regulates breast cancer tumourigenesis *in vivo*. Previous studies demonstrated that transient inhibition of Nodal with Morpholinos or exposure to its antagonist, Lefty, diminished tumour initiation in breast cancer and melanoma models [9], [11]. In order to better understand the role of Nodal in tumour growth over an extended period of time, we stably knocked down Nodal expression in aggressive MDA-MB-468 and MDA-MB-231 breast cancer cells using puromycin-selectable shRNAs. In our first model, we injected 2.5 million MDA-MB-468 cells transfected with a Control shRNA (468+shControl) or a Nodal-targeted shRNA (468+shNodal) into the flanks of nude mice, and measured tumour growth over 6 weeks. This approach revealed that Nodal knockdown significantly impaired MDA-MB-468 tumour growth, and resulted in>2-fold reduction in tumour volume following excision (p<0.05) (**Fig. 2A–C**).

As a corollary to this experiment, in our second model, we injected 500,000 MDA-MB-231 cells transfected with a Control shRNA (231+shControl) or Nodal-targeted shRNA (231+shNodal) through the nipple into the mammary fat pad of nude mice, and measured tumour growth over 9 weeks. Compared to our flank model, this model was more stringent since we injected fewer cells (0.5 versus 2.5 million), Matrigel was not used to help tumours initiate, and the breast cancer cells were injected into the mammary environment to recapitulate a relevant physiological context. We found that Nodal knockdown significantly impaired tumour growth compared to controls (p<0.05, n = 10) (**Fig. 2D,E**). Furthermore, there was a 5-fold reduction in tumour volume following excision (p<0.05) (**Fig. 2F**). Importantly, we observed a phenomenon that was not apparent in our flank model. We found that unlike 231+shControl tumours which continued to grow over time, the 231+shNodal tumours experienced a plateau in growth at a diameter of approximately 1.5 mm. This suggested to us that Nodal inhibition may alter proliferation and cell death ratios to counteract tumour growth.

### Loss of Nodal Expression Reduces Proliferation and Increases Apoptotic Phenotypes in Aggressive MDA-MB-231 Breast Cancer Cells

**Figure 3:**
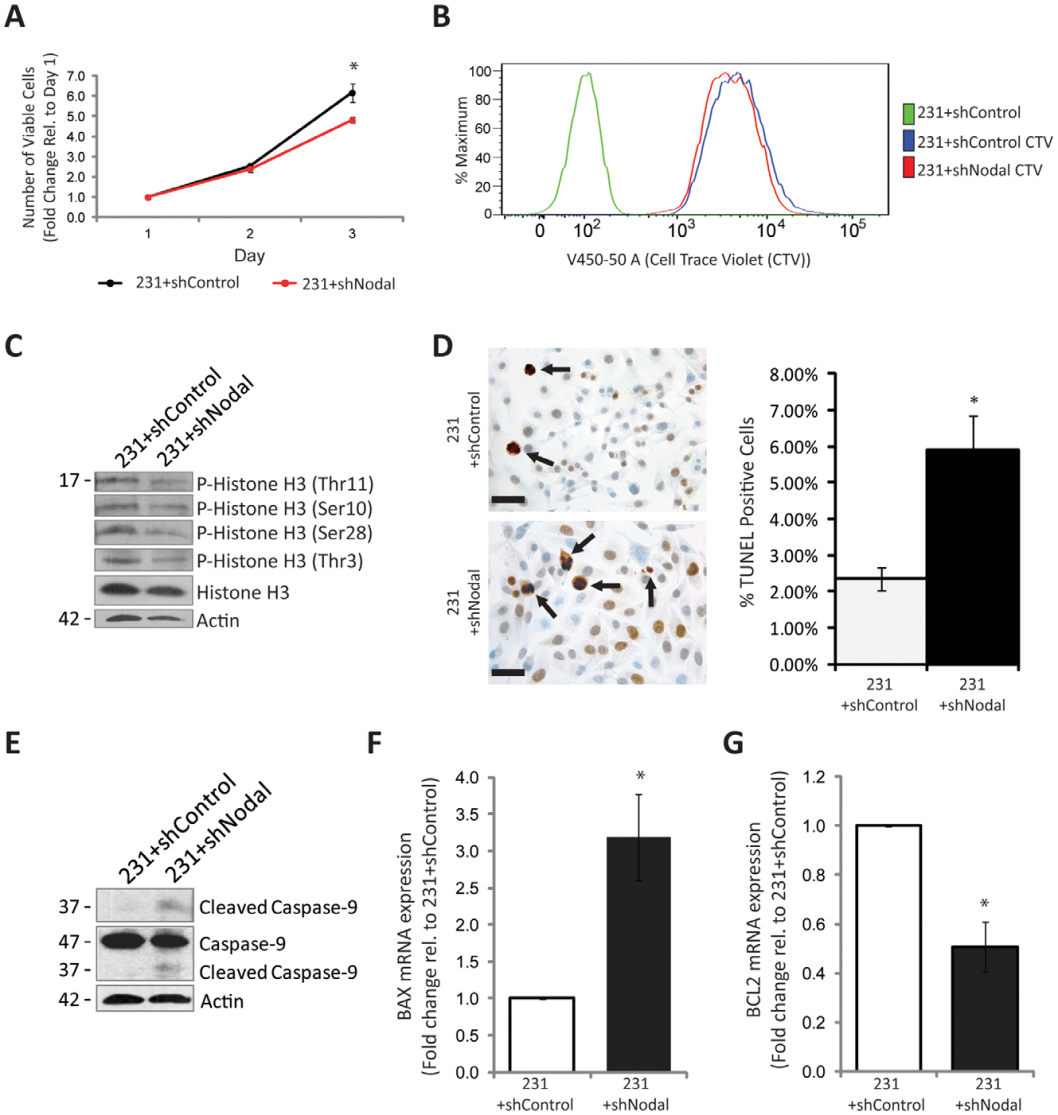
Nodal knockdown decreases proliferation and increases apoptosis in aggressive MDA-MB-231 breast cancer cells. (A) Trypan Blue exclusion was used to count live cells daily to generate growth curves over 3 days, in response to altered Nodal expression. MDA-MB-231 cells transfected with a Nodal-targeted shRNA (231+shNodal) exhibited a significant decrease in proliferation over 3 days compared to cells transfected with a scrambled Control shRNA (231+shControl) (n = 3; p = 0.047). (B) Representative histogram of mean fluorescence intensity (mfi) in 231+shNodal and 231+shControl cells labelled with Cell Trace Violet (CTV) for 4 days after synchronization via serum starvation. There was a greater loss of CTV in 231+shControl compared to 231+shNodal cells (4801 and 6006, respectively) indicating that control cells proliferated more than their shNodal-treated counterparts. (C) Western blots demonstrating decreased phosphorylated histone H3 at 4 different sites, including Thr11, Ser10, Ser28 and Thr3 in 231+shNodal cells compared to 231+shContol cells. Total histone H3 and β-Actin are used as controls. (D) Representative images of TUNEL staining and corresponding quantification of percent TUNEL-positive cells in 231+shNodal and 231+shControl cells grown *in vitro*. TUNEL positive cells are delineated by arrows and nuclei are counterstained blue. Micron bars equal 25 µm. The percentage of TUNEL positive cells is significantly higher in 231+shNodal cells as compared to 231+shControl cells (n = 23; p = 0.001). (E) Western blot demonstrating that cleavage of caspase-9 is elevated in 231+shNodal compared to 231+shControl cells. Uncleaved caspase-9 and β-Actin are used as controls. (F) Real time RT-PCR analysis demonstrating that *BAX* mRNA expression is significantly higher in 231+shNodal cells compared to 231+shControl cells (n = 4, p = 0.029). (G) Real time RT-PCR analysis demonstrating that *BCL2* mRNA expression is significantly lower in 231+shNodal cells compared to 231+shControl cells (n = 4, p = 0.029). All bar graphs are presented as mean ± S.E.M. for replicate values. Asterisks indicate a significant difference as specified compared to controls.

Given our finding that Nodal inhibition causes a reduction in tumour growth *in vivo,* we examined the effects of Nodal knockdown on cell proliferation *in vitro* using Trypan Blue exclusion assays. As a Nodal loss-of-function model, we compared growth curves for 231+shNodal cells versus 231+shControl cells. We found that Nodal inhibition by shRNA significantly reduced the number of viable cells in MDA-MB-231 cells (n = 3, p = 0.047) compared to controls after 3 days (**Fig. 3A**). In addition, we labelled 231+shNodal and 231+shControl cells with Cell Trace Violet (CTV) after synchronization via serum starvation and then quantified CTV loss as a measure of proliferation. A representative example of three independent experiments is shown in **Fig. 3B**. After four days, determination of mean fluorescence intensity via flow cytometry showed a greater loss of CTV in 231+shControl compared to 231+shNodal cells (4801 and 6006, respectively) indicating that shControl cells proliferated more than their shNodal counterparts (**Fig. 3B**). In accordance with these results, Western blot analyses revealed that Histone H3 phosphorylation was lower in 231+shNodal cells compared to 231+shControl cells, indicative of reduced mitosis (**Fig. 3C**). Taken together, Nodal loss-of-function decreased proliferation; however the changes were small, suggesting that alterations in proliferation did not solely account for the robust effects seen on tumour growth *in vivo.*

Given Nodal had a small effect on proliferation *in vitro,* we hypothesized that perhaps Nodal could also regulate apoptotic phenotypes, which might help to explain the large differences observed during *in vivo* tumour growth. In order to explore the role of Nodal in the regulation of apoptosis, we first examined the effects of knocking Nodal down on the percentage of TUNEL positive (apoptotic) cells *in vitro*. Accordingly, Nodal knockdown in MDA-MB-231 cells resulted in a 3.5% increase (from 2.4 to 5.9%) in the number of apoptotic cells as compared to control conditions (n = 24, p = 0.001) (**Fig. 3D**). Furthermore, Western blot analyses for activated (cleaved) caspase-9 in 231+shNodal cells versus 231+shControl cells revealed that cleaved caspase-9 is increased in response to Nodal knockdown, indicative of elevated levels of apoptosis (**Fig. 3E**). Given that caspase-9 is frequently associated with mitochondria-mediated apoptosis, we quantified *BAX* and *BCL2* mRNA expression in 231+Control cells versus 231+shNodal cells, since these factors also play a role in mitochondria-mediated apoptosis. Real time RT-PCR analysis indicated that there was a significant increase in *BAX* expression (n = 4, p = 0.029) and a significant decrease in *BCL2* expression (n = 4, p = 0.029) in 231+shNodal cells compared to controls **(** **Fig. 3F,G** **).**

### Gain of Nodal Expression Increases Proliferation and Decreases Apoptotic Phenotypes in Poorly Aggressive T47D Breast Cancer Cells

**Figure 4:**
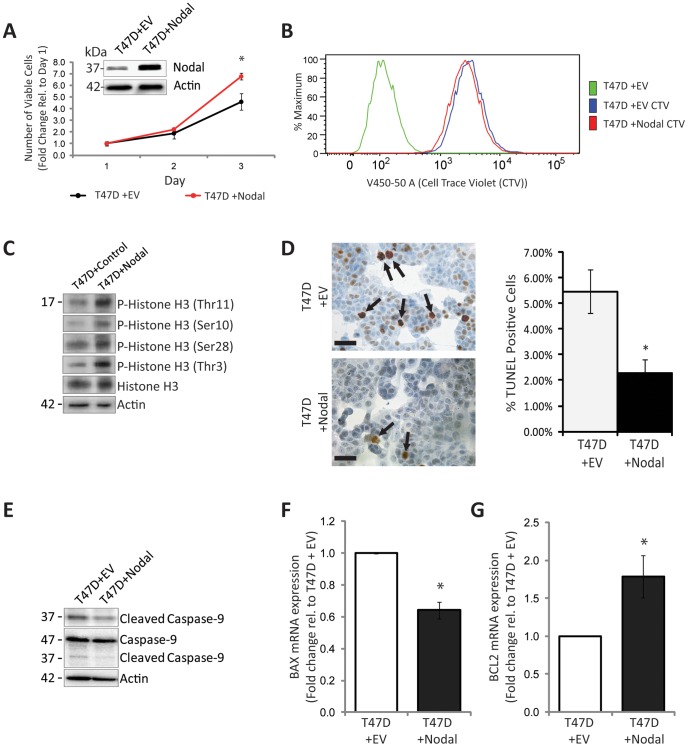
Nodal over-expression increases proliferation and decreases apoptosis in poorly aggressive T47D breast cancer cells. (A) Western blot demonstrating that Nodal protein is elevated in T47D cells transfected with a Nodal expression construct (T47D+Nodal) compared to empty vector controls (T47D+EV). The ∼39 kDa Pro-Nodal band is presented and Actin is used as a loading control. Trypan Blue exclusion was used to count live cells daily to generate growth curves over 3 days, in response to altered Nodal expression. T47D+Nodal cells exhibited a significant increase in proliferation compared to T47D+EV cells over 3 days (n = 3, p = 0.046). (B) Representative histogram of mfi in T47D+EV and T47D+Nodal cells labelled with CTV for 6 days after synchronization via serum starvation. There was a greater loss of CTV in T47D+Nodal compared to T47D+EV cells (2738 and 3319, respectively) indicating that proliferation increased with Nodal over-expression. (C) Western blots demonstrating increased phosphorylated histone H3 at 4 different sites, including Thr11, Ser10, Ser28 and Thr3 in T47D+Nodal cells compared to T47D+EV cells. Total histone H3 and β-Actin are used as controls. (D) Representative images of TUNEL staining and corresponding quantification of percent TUNEL-positive cells in T47D+EV and T47D+Nodal cells grown *in vitro*. TUNEL positive cells are delineated by arrows and nuclei are counterstained blue. Micron bars equal 25 µm. The percentage of TUNEL positive cells is significantly higher in T47D+EV cells as compared to T47D+Nodal cells (n = 8; p = 0.006). (E) Western blot demonstrating that cleavage of caspase-9 is reduced in T47D+Nodal cells compared to T47D+EV cells. Uncleaved caspase-9 and β-Actin are used as controls. (F) Real time RT-PCR analysis demonstrating that *BAX* mRNA expression is significantly lower in T47D+Nodal cells compared to T47D+EV cells (n = 5, p = 0.016). (G) Real time RT-PCR analysis demonstrating that *BCL2* mRNA expression is significantly higher in T47D+Nodal cells compared to T47D+EV cells (n = 5, p = 0.016). All data are presented as mean ± S.E.M. for replicate values. Asterisks indicate a significant difference compared to controls.

As an extension to our loss-of-function results, we next examined the effects of over-expressing Nodal in poorly aggressive T47D breast cancer cells (T47D+Nodal), which do not normally express high levels of this morphogen, versus transfection with an empty vector control (T47D+EV). Using a trypan blue exclusion assay, we found that T47D+Nodal cells displayed a significant increase in proliferation after 3 days compared to T47D+EV cells (n = 3; p = 0.046) (**Fig. 4A**). Likewise, using the CTV assay in three independent experiments, we determined that T47D+Nodal cells proliferated more than parental T47D+EV cells as evidenced by lower CTV mfi (**Fig. 4B**). In the representative example shown, CTV mfi was 2738 for T47D+Nodal compared to 3319 for T47D+EV cells six days post-synchronization. When Western blot analysis was performed to evaluate the phosphorylation status of Histone H3, we found that T47D+Nodal cells displayed elevated P-Histone H3 at four different sites (including Ser10, Ser28, Thr3, and Thr11) compared to T47D+EV cells, indicative of elevated mitosis (**Fig. 4C**). Of note, using a fluorescence-based Live-Dead assay, in a previous study we did not see major changes in cell viability in T47D+Nodal cells relative to T47D+EV cells [21]. This was likely because the Live/Dead assay was less sensitive than the multiple assays employed here.

To determine the effects of Nodal on apoptosis, we first conducted TUNEL staining on cells grown *in vitro*. In accordance with our loss-of-function data, Nodal over-expression in T47D cells resulted in a 3.2% decrease (from 5.5 to 2.3%) in the number of apoptotic cells as compared to control conditions (n = 8, p = 0.006) (**Fig. 4D**). In order to explore the effects of Nodal on apoptosis, we performed Western blot analyses for activated (cleaved) caspase-9 in T47D+EV cells versus T47D+Nodal cells. We found that cleaved caspase-9 was present at lower levels in T47D+Nodal cells compared controls, indicative of reduced apoptosis in the presence of Nodal (**Fig. 4E**). Accordingly, real time RT-PCR analysis indicated that there was a significant decrease in *BAX* expression (n = 5, p = 0.016) and a significant increase in *BCL2* expression (n = 5, p = 0.016) in T47D+Nodal cells compared to controls **(** **Fig. 4F,G** **).**

Taken together, our results suggest that Nodal promotes the net growth of breast cancer cells in culture by increasing proliferation and decreasing apoptosis. This in part explains the observation that Nodal inhibition blunts tumour growth *in vivo*. Interestingly, it has been reported that Notch4, which regulates Nodal expression in melanoma models, promotes proliferation and inhibits apoptosis in C8161, MV3, and SK-MEL-28 melanoma cell lines [28]. A recent study also demonstrated that blocking Nodal signaling with a function-blocking antibody decreases proliferation and increases apoptosis of MDA-MB-231 and MDA-MB-468 cells *in vitro* [18], supporting the results presented here. Furthermore, Nodal over-expression in GBM glioma cells causes an increase in proliferation concomitant with elevated tumourigenesis in mice [8]. In contrast to the results shown here, it has been reported that over-expression of Nodal promotes apoptosis and inhibits proliferation in MDA-MB-231 breast cancer cell lines [29], [30]. However, one key difference in this study is that Nodal was overexpressed in MDA-MB-231 cells (which we have shown express high endogenous Nodal), whereas in the current study, Nodal was inhibited in MDA-MB-231 cells. Furthermore, the dose of recombinant mouse Nodal that was used in the previous study was 500 ng/mL, which is 5-fold higher than the dose of recombinant human Nodal used in this investigation [30]. This brings light to the possibility that Nodal exhibits a concentration-dependent biphasic effect on breast cancer progression, similar to the function of TGF-β [31], [32].

### Inhibition of ALK4/7 (Nodal Type I Receptor) Reduces Proliferation and Increases Apoptotic Phenotypes in Highly Aggressive Breast Cancer Cell Lines

**Figure 5:**
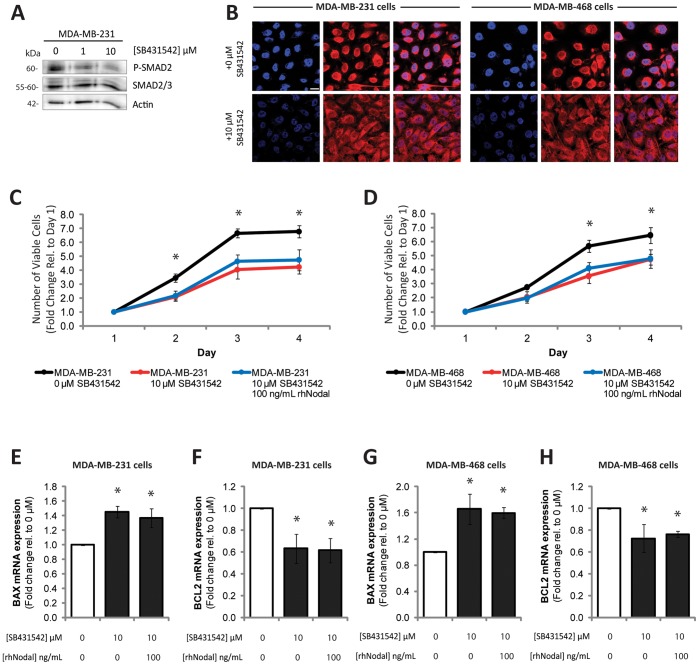
The effects of Nodal on proliferation and apoptosis are dependent on activation of the type 1 receptor (ALK4/7). (A) Western blot analysis demonstrating that phosphorylation of SMAD2 decreases with SB431542 treatment (1–10 µM) in MDA-MB-231 cells. SMAD2/3 and Actin are used as loading controls. (B) Immunofluorescence localization of SMAD2/3 (red) demonstrating that in MDA-MB-231 cells and in MDA-MB-468 cells, SMAD2/3 localization to the nucleus is reduced upon treatment with SB431542 (10 µM). DAPI (blue) is used to stain nuclei and bar equals 10 µm. (C) Growth curves for MDA-MB-231 cells or (D) MDA-MB-468 cells over 4 days demonstrating that inhibition of the Nodal type 1 receptor (ALK4/7) with SB431542 (10 µM) causes reduced growth over time, and this is not rescued with addition of rhNodal (100 ng/mL). Time points indicated by an asterisk (*) demonstrated a significant difference between controls and all other treatments. (E) PCR analysis demonstrating that treatment of MDA-MB-231 cells with 10 µM SB431542 causes a significant increase in *BAX* expression (n = 3, p = 0.002), and (F) a significant decrease in *BCL2* expression (n = 3, p = 0.022), indicative of elevated apoptosis compared to vehicle controls. Recombinant human Nodal (100 ng/mL) was used in combination with SB431542, and no rescue in either *BAX* or *BCL2* expression was observed. (G) PCR analysis demonstrating that treatment of MDA-MB-468 cells with 10 µM SB431542 causes a significant increase in *BAX* expression (n = 3, p = 0.020), and (H) a significant decrease in *BCL2* expression (n = 3, p = 0.046) compared to vehicle controls. Recombinant human Nodal (100 ng/mL) was used in combination with SB431542, and no rescue in either *BAX* or *BCL2* expression was observed. Data are presented as mean ± S.E.M. for replicate values. All experiments were repeated ≥3 times. Values indicated by an asterisk (*) are significantly different from controls.

Nodal’s canonical signalling pathway includes binding to a receptor complex (including ALK4/7, ActRIIB, and Cripto) to activate phosphorylation of SMAD2/3, which translocates to the nucleus with SMAD4 to regulate gene transcription. To determine whether this receptor complex is important for cellular growth, we used a small molecule inhibitor, SB431542, which blocks the Nodal type 1 receptor (ALK4/7), and evaluated the effects of rhNodal treatment on cellular growth, and *BAX* and *BCL2* expression. We first validated that treatment with SB431542 reduced phosphorylation of SMAD2 in MDA-MB-231 cell lines (which express high levels of Nodal) (**Fig. 5A**). We also performed immunofluorescence for SMAD2/3 in MDA-MB-231 and MDA-MB-468 cell lines, to validate that treatment with SB431542 caused reduced nuclear translocation of SMAD2/3, which is indicative of decreased SMAD activation (**Fig. 5B**). We found that treatment of both cell lines with 10 µM SB431542 caused reduced cellular growth over the course of 4 days, and this effect was not rescued by addition of rhNodal (**Fig. 5C,D**), suggesting that Nodal needs to activate the ALK4/7 receptor to increase proliferation. Furthermore, we found that treatment of MDA-MB-231 cells with 10 µM SB431542 caused a significant increase in *BAX* expression (n = 3, p = 0.002), and a significant decrease in *BCL2* expression (n = 3, p = 0.022), indicative of elevated apoptosis compared to vehicle controls (**Fig. 5E,F**). Similarly, treatment of MDA-MB-468 cells with 10 µM SB431542 caused a significant increase in *BAX* expression (n = 3, p = 0.020), and a significant decrease in *BCL2* expression (n = 3, p = 0.046) (**Fig. 5G,H**). Addition of rhNodal to SB431542-treated MDA-MB-231 cells or MDA-MB-468 cells did not rescue *BAX* or *BCL2* expression in either cell line, indicating that regulation of *BAX* and *BCL2* gene expression is dependent on activation of the Nodal type 1 receptor (**Fig. 5E-H**).

To our knowledge, no other receptors (besides the ALK4/7/ActRIIB/Cripto receptor complex) have been shown to interact with Nodal; therefore, we suspect that Nodal mediates its mitogenic effects through its canonical SMAD pathway. However, it remains elusive whether Nodal can elicit its effects through a non-canonical mechanism. For example, we have shown that Nodal is capable of mediating phosphorylation of ERK1/2 to promote invasive phenotypes in breast cancer and choriocarcinoma cell lines. Given that ERK signalling is frequently associated with elevated mitogenic activity in cancer, studies geared towards exploring whether ERK signalling is involved in the Nodal-induced phenotypes presented here would be of interest to pursue in the future. Finally, it should be noted that SB431542 does not selectively inhibit Nodal signaling: It can also block Activin and TGF-β induced responses [22]. Hence it is also possible, that Nodal mediates its effects on proliferation by regulating these pathways.

### Nodal Promotes Growth from Micro to Macrometastases

Cancer becomes a fatal disease once it has metastasized and grown into a sufficient secondary tumour mass. However, the metastatic cascade is a highly inefficient process overall, and it has been reported that one of the most inefficient steps is growth at the secondary site [33], [34]. Given that Nodal inhibition causes a plateau in primary tumour growth *in vivo*, and that it alters proliferation and cell death *in vitro*, we opted to test the effect of Nodal inhibition on secondary tumour growth.

**Figure 6:**
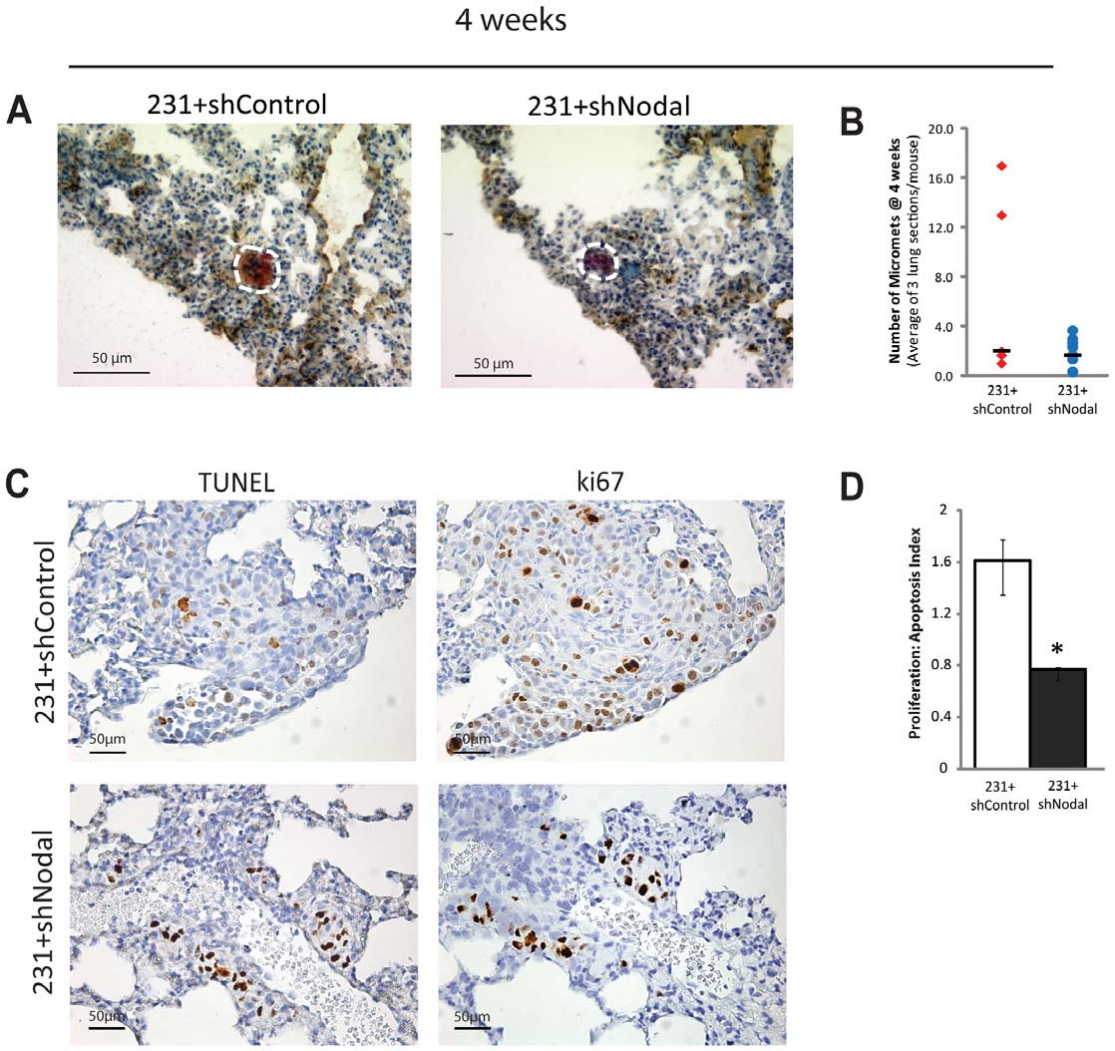
Nodal inhibition alters proliferation:apoptosis ratios in micrometastases. (A) GUSB staining of pulmonary micrometastases from MDA-MB-231 cells transfected with a Control shRNA (231+shControl) or a shRNA to Nodal (231+shNodal) in NOD/SCID/MPSVII mice 4 weeks post-intravenous injection (red and outlined with white dotted line). (B) Scatter plot representing the average number of micrometastases (<100 cells) per section of lung from NOD/SCID/MPSVII mice 4 weeks after injection with 231+shControl or 231+shNodal cells. The number of 231+shNodal micrometastases that formed after 4 weeks in NOD/SCID/MPSVII mice was not significantly reduced compared to the number of 231+shControl micrometastases (n≥5, p>0.05). Each point represents the average mean number of micrometastases per section per mouse. Black bars represent the median number of micrometastases per section per mouse. (C) Immunohistochemical analysis of Ki67 expression (brown) and TUNEL (brown) staining in pulmonary micrometastases from 231+shControl cells or 231+shNodal cells in NOD/SCID/MPSVII mice 4 weeks post-intravenous injection. Proliferation is indicated by Ki67 staining and apoptotic nuclei were detected with TUNEL. (D) Proliferation:apoptosis ratios in 4 week micrometastases were determined with immunohistochemical localization of Ki67 and TUNEL. At 4 weeks, lesions from 231+shControl cells had a positive proliferation ratio (1.57) whereas lesions from 231+shNodal cells had a negative proliferation:apoptosis ratio (0.74) (n≥3, p<0.05). Values represent mean average proliferation:apoptosis ratio in tumour lesions per mouse ± S.E.M.

Accordingly, we developed a model that takes advantage of a simple experimental metastasis assay using beta-glucuronidase (GUSB)-deficient NOD/SCID/mucopolysaccharidosis type VII (NOD/SCID) mice [25]. The GUSB model allowed us to attain single-cell resolution of transplanted human tumour cells by virtue of their constitutive GUSB activity within the GUSB-deficient mouse, thereby enabling the identification of lesions down to single-cell level that would be undetectable using conventional histology. We sacrificed the mice at two different time points, at 4 weeks and 8 weeks, following tail vein injection of 231+shControl or 231+shNodal cells. At both 4 and 8 weeks, brain and liver tissue were also evaluated for evidence of metastasis. Using this high-resolution experimental metastasis model, we found only micrometastases of approximately 1–100 cells at 4 weeks post-injection, and discovered that Nodal knockdown did not cause a significant change in the number of micrometastases that formed in the lung (**Fig. 6A,B**). This suggested that Nodal does not affect seeding at secondary sites.

In addition to seeding, another important step in the metastatic cascade is growth at the secondary site. Indeed, metastatic tumour cells can reside and survive at secondary sites in the body while circumventing a need for growth or progression; an aspect of carcinogenesis called tumour dormancy. Tumour mass dormancy, in particular, refers to metastases that remain asymptomatic due to an inability to expand in size, and is often attributed to a counterbalance of proliferation and apoptosis (reviewed in [35]). Dormant tumours are often not dangerous; however, their potential to overcome their dormant state poses a threat to patient health. Accordingly, we measured proliferation-to-apoptosis ratios in the 4-week micro-lesions via immunohistochemical staining for Ki67:TUNEL. We found that 231+shControl lesions had a proliferation-to-apoptosis ratio greater than 1, indicating a potential for tumour growth, whereas 231+shNodal lesions had a proliferation-to-apoptosis ratio less than 1, indicating a state of tumour mass dormancy or regression (**Fig. 6C,D**).

**Figure 7:**
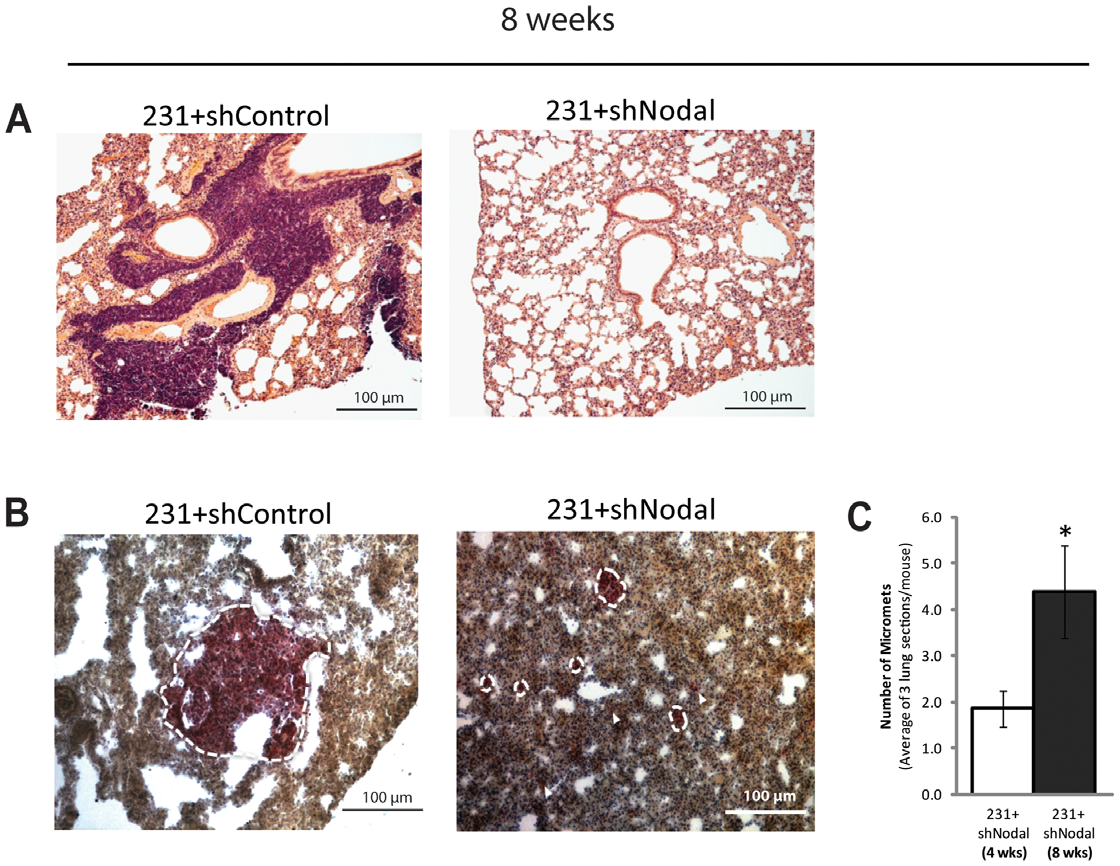
Nodal knockdown mitigates progression to macrometastases. (A) H&E staining demonstrates macrometastasis formation after 8 weeks post-intravenous injection in NOD/SCID/MPSVII mice for 231+shControl cells, but not 231+shNodal cells. 100% of lungs seeded with 231+shControl cells (4/4) contained macroscopic lesions at 8 weeks, whereas 0/8 lungs seeded with 231+shNodal cells contained macroscopic lesions. (B) GUSB staining to confirm human origin of lesions showing 231+shControl macrometastasis formation after 8 weeks. Although macrometastases were not detected in mice injected with 231+shNodal cells, micrometastases were detected with GUSB staining. (C) The number of 231+shNodal micrometastases that formed after 8 weeks in NOD/SCID/MPSVII mice was significantly higher than those that formed after 4 weeks (n = 8, p<0.05). Bars represent the average mean number of micrometastases per section per mouse ± S.E.M.

Given these results, we expected that tumours that exhibited a potential for growth at 4 weeks would progress to macrometastases by 8 weeks, and far exceed the 100-cell limit observed in the 4-week lesions. Of note, by 8 weeks the 231+shControl lesions formed overt pulmonary metastases in all of the mice injected with these cells (**Fig. 7A,B**). In contrast, the 231+shNodal cells did not form macrometastases. Rather, at 8 weeks there was a significant accumulation of micrometastases in the lung compared to 231+shNodal cells at the 4 week time point (n≥8, p<0.05) (**Fig. 7C**). We also detected metastases in the brain of 1/5 231+shControl injected mice at 4 weeks and in the liver of 1/4 231+shControl injected mice at 8 weeks. However, metastases to the brain or the liver were not detected in any of the 16 231+shNodal-injected mice (**Fig. S1A,B**).

Taken together, the results from our experimental metastasis assay illustrate the importance of Nodal in regulating the transition between micrometastatic and macrometastatic growth, in part through its ability to alter proliferation-to-apoptosis ratios necessary for normal tissue homeostasis. Similar phenomena have been reported in C57BL6/J mouse models of Lewis lung carcinoma mice, whereby poor tumour vascularization caused tumour dormancy marked by equal rates of mitosis and apoptosis [36]. Interestingly, previous findings from our laboratory have implicated Nodal in regulating breast cancer angiogenesis both *in vitro* and *in vivo* [21]. Thus in addition to directly regulating cell proliferation and/or apoptosis, Nodal may indirectly promote tumour growth by facilitating vascular recruitment. Finally, although Nodal did not affect seeding of the cells at the secondary site, micrometastases accumulated over time with Nodal-deficient cells. Conventional tumour growth assays using histology and/or whole animal imaging do not permit the single cell resolution that we obtained with the GUSB model; hence, studies done using conventional methodologies may inadvertently overlook the seeding phenomenon uncovered here.

### Conclusions

Collectively, this study indicates that the stem cell-associated protein Nodal promotes breast cancer tumour growth at both primary and secondary tumour sites, by altering the balance between proliferation and apoptosis. Our results provide mechanistic insight into studies that demonstrate that cancer cells manifesting stem-cell like properties exhibit accelerated cancer growth and progression *in vivo*, compared to well-differentiated counterparts [37], [38]. Since Nodal expression is limited to embryonic contexts, our discovery suggests a novel role for Nodal as a tumour-specific target against breast cancer progression, and for maintenance of tumour dormancy following metastatic spread.

## Supporting Information

Figure S1 **Nodal supports tumour metastasis.** (A) GUSB staining of a brain metastasis from MDA-MB-231 cells transfected with a Control shRNA (231+shControl) in NOD/SCID/MPSVII mice 4 weeks post-intravenous injection (red). (B) GUSB staining demonstrates a liver macrometastasis from 231+shControl cells after 8 weeks. No tumours were found in either brain or liver from 231+shNodal cells at either 4 or 8 weeks.
(PDF)

Table S1 (PDF)

Table S2 (PDF)

## References

[1] Hanahan D, Weinberg RA (2011) Hallmarks of cancer: the next generation. Cell 144: 646–674. 21376230 10.1016/j.cell.2011.02.013

[2] Hanahan D, Weinberg RA (2000) The hallmarks of cancer. Cell 100: 57–70. 10647931 10.1016/s0092-8674(00)81683-9

[3] Yerushalmi R, Woods R, Ravdin PM, Hayes MM, Gelmon KA (2010) Ki67 in breast cancer: prognostic and predictive potential. Lancet Oncol 11: 174–183. 20152769 10.1016/S1470-2045(09)70262-1

[4] Urruticoechea A, Smith IE, Dowsett M (2005) Proliferation marker Ki-67 in early breast cancer. J Clin Oncol 23: 7212–7220. 16192605 10.1200/JCO.2005.07.501

[5] Dowsett M, Nielsen TO, A'Hern R, Bartlett J, Coombes RC, et al (2011) Assessment of Ki67 in breast cancer: recommendations from the International Ki67 in Breast Cancer working group. J Natl Cancer Inst 103: 1656–1664. 21960707 10.1093/jnci/djr393 PMC3216967

[6] Vermeulen PB, Gasparini G, Fox SB, Toi M, Martin L, et al (1996) Quantification of angiogenesis in solid human tumours: an international consensus on the methodology and criteria of evaluation. Eur J Cancer 32A: 2474–2484. 9059336 10.1016/s0959-8049(96)00379-6

[7] Lawrence MG, Margaryan NV, Loessner D, Collins A, Kerr KM, et al (2011) Reactivation of embryonic nodal signaling is associated with tumor progression and promotes the growth of prostate cancer cells. Prostate 71: 1198–1209. 21656830 10.1002/pros.21335 PMC3234312

[8] Lee CC, Jan HJ, Lai JH, Ma HI, Hueng DY, et al (2010) Nodal promotes growth and invasion in human gliomas. Oncogene 29: 3110–3123. 20383200 10.1038/onc.2010.55

[9] Topczewska JM, Postovit LM, Margaryan NV, Sam A, Hess AR, et al (2006) Embryonic and tumorigenic pathways converge via Nodal signaling: role in melanoma aggressiveness. Nat Med 12: 925–932. 16892036 10.1038/nm1448

[10] Postovit LM, Seftor EA, Seftor RE, Hendrix MJ (2007) Targeting Nodal in malignant melanoma cells. Expert Opin Ther Targets 11: 497–505. 17373879 10.1517/14728222.11.4.497

[11] Postovit LM, Margaryan NV, Seftor EA, Kirschmann DA, Lipavsky A, et al (2008) Human embryonic stem cell microenvironment suppresses the tumorigenic phenotype of aggressive cancer cells. Proc Natl Acad Sci U S A 105: 4329–4334. 18334633 10.1073/pnas.0800467105 PMC2393795

[12] Strizzi L, Postovit LM, Margaryan NV, Lipavsky A, Gadiot J, et al (2009) Nodal as a biomarker for melanoma progression and a new therapeutic target for clinical intervention. Expert Rev Dermatol 4: 67–78. 19885369 10.1586/17469872.4.1.67 PMC2682534

[13] Papageorgiou I, Nicholls PK, Wang F, Lackmann M, Makanji Y, et al (2009) Expression of nodal signalling components in cycling human endometrium and in endometrial cancer. Reprod Biol Endocrinol 7: 122. 19874624 10.1186/1477-7827-7-122 PMC2774317

[14] Lonardo E, Hermann PC, Mueller MT, Huber S, Balic A, et al (2011) Nodal/Activin signaling drives self-renewal and tumorigenicity of pancreatic cancer stem cells and provides a target for combined drug therapy. Cell Stem Cell 9: 433–446. 22056140 10.1016/j.stem.2011.10.001

[15] Cavallari C, Fonsato V, Herrera MB, Bruno S, Tetta C, et al.. (2012) Role of Lefty in the anti tumor activity of human adult liver stem cells. Oncogene: onc2012114 [pii];10.1038/onc.2012.114 [doi]. 10.1038/onc.2012.114 22469982

[16] Kenney NJ, Adkins HB, Sanicola M (2004) Nodal and Cripto-1: embryonic pattern formation genes involved in mammary gland development and tumorigenesis. J Mammary Gland Biol Neoplasia 9: 133–144. 15300009 10.1023/B:JOMG.0000037158.91940.1c

[17] Bianco C, Adkins HB, Wechselberger C, Seno M, Normanno N, et al (2002) Cripto-1 activates nodal- and ALK4-dependent and -independent signaling pathways in mammary epithelial Cells. Mol Cell Biol 22: 2586–2597. 11909953 10.1128/MCB.22.8.2586-2597.2002 PMC133714

[18] Strizzi L, Hardy KM, Margaryan NV, Hillman DW, Seftor EA, et al (2012) Potential for the embryonic morphogen Nodal as a prognostic and predictive biomarker in breast cancer. Breast Cancer Res 14: R75. 22577960 10.1186/bcr3185 PMC3446338

[19] Strizzi L, Postovit LM, Margaryan NV, Seftor EA, Abbott DE, et al (2008) Emerging roles of nodal and Cripto-1: from embryogenesis to breast cancer progression. Breast Dis 29: 91–103. 19029628 10.3233/bd-2008-29110 PMC3175751

[20] Kao J, Salari K, Bocanegra M, Choi YL, Girard L, et al (2009) Molecular profiling of breast cancer cell lines defines relevant tumor models and provides a resource for cancer gene discovery. PLoS One 4: e6146. 19582160 10.1371/journal.pone.0006146 PMC2702084

[21] Quail DF, Walsh LA, Zhang G, Findlay SD, Moreno J, et al (2012) Embryonic Protein Nodal Promotes Breast Cancer Vascularization. Cancer Research 72: 3851–3863. 22855743 10.1158/0008-5472.CAN-11-3951

[22] Inman GJ, Nicolas FJ, Callahan JF, Harling JD, Gaster LM, et al (2002) SB-431542 is a potent and specific inhibitor of transforming growth factor-beta superfamily type I activin receptor-like kinase (ALK) receptors ALK4, ALK5, and ALK7. Mol Pharmacol 62: 65–74. 12065756 10.1124/mol.62.1.65

[23] Le Good JA, Joubin K, Giraldez AJ, Ben-Haim N, Beck S, et al (2005) Nodal stability determines signaling range. Curr Biol 15: 31–36. 15649361 10.1016/j.cub.2004.12.062

[24] Quail DF, Taylor MJ, Walsh LA, Dieters-Castator D, Das P, et al (2011) Low oxygen levels induce the expression of the embryonic morphogen Nodal. Mol Biol Cell 22: 4809–4821. 22031289 10.1091/mbc.E11-03-0263 PMC3237624

[25] Hess DA, Craft TP, Wirthlin L, Hohm S, Zhou P, et al (2008) Widespread nonhematopoietic tissue distribution by transplanted human progenitor cells with high aldehyde dehydrogenase activity. Stem Cells 26: 611–620. 18055447 10.1634/stemcells.2007-0429 PMC3045698

[26] Schier AF (2009) Nodal morphogens. Cold Spring Harb Perspect Biol 1: a003459. 20066122 10.1101/cshperspect.a003459 PMC2773646

[27] Vo BT, Khan SA (2011) Expression of nodal and nodal receptors in prostate stem cells and prostate cancer cells: autocrine effects on cell proliferation and migration. Prostate 71: 1084–1096. 21557273 10.1002/pros.21326 PMC3139718

[28] Hardy KM, Kirschmann DA, Seftor EA, Margaryan NV, Postovit LM, et al (2010) Regulation of the embryonic morphogen Nodal by Notch4 facilitates manifestation of the aggressive melanoma phenotype. Cancer Res 70: 10340–10350. 21159651 10.1158/0008-5472.CAN-10-0705 PMC3057934

[29] Xu G, Zhong Y, Munir S, Yang BB, Tsang BK, et al (2004) Nodal induces apoptosis and inhibits proliferation in human epithelial ovarian cancer cells via activin receptor-like kinase 7. J Clin Endocrinol Metab 89: 5523–5534. 15531507 10.1210/jc.2004-0893

[30] Zhong Y, Xu G, Ye G, Lee D, Modica-Amore J, et al (2009) Nodal and activin receptor-like kinase 7 induce apoptosis in human breast cancer cell lines: Role of caspase 3. Int J Physiol Pathophysiol Pharmacol 1: 83–96. 21383881 PMC3040937

[31] Bachman KE, Park BH (2005) Duel nature of TGF-beta signaling: tumor suppressor vs. tumor promoter. Curr Opin Oncol 17: 49–54. 15608513 10.1097/01.cco.0000143682.45316.ae

[32] Pardali K, Moustakas A (2007) Actions of TGF-beta as tumor suppressor and pro-metastatic factor in human cancer. Biochim Biophys Acta 1775: 21–62. 16904831 10.1016/j.bbcan.2006.06.004

[33] Luzzi KJ, MacDonald IC, Schmidt EE, Kerkvliet N, Morris VL, et al (1998) Am J Pathol. 153: 865–873. 10.1016/S0002-9440(10)65628-3 PMC1853000 9736035

[34] Hunter KW, Crawford NP, Alsarraj J (2008) Mechanisms of metastasis. Breast Cancer Res 10 Suppl 1 S2. 10.1186/bcr1988 PMC2605099 19091006

[35] Aguirre-Ghiso JA (2007) Models, mechanisms and clinical evidence for cancer dormancy. Nat Rev Cancer 7: 834–846. 17957189 10.1038/nrc2256 PMC2519109

[36] Holmgren L, O'Reilly MS, Folkman J (1995) Dormancy of micrometastases: balanced proliferation and apoptosis in the presence of angiogenesis suppression. Nat Med 1: 149–153. 7585012 10.1038/nm0295-149

[37] Al-Hajj M, Wicha MS, ito-Hernandez A, Morrison SJ, Clarke MF (2003) Prospective identification of tumorigenic breast cancer cells. Proc Natl Acad Sci U S A 100: 3983–3988. 12629218 10.1073/pnas.0530291100 PMC153034

[38] Croker AK, Goodale D, Chu J, Postenka C, Hedley BD, et al (2009) High aldehyde dehydrogenase and expression of cancer stem cell markers selects for breast cancer cells with enhanced malignant and metastatic ability. J Cell Mol Med 13: 2236–2252. 18681906 10.1111/j.1582-4934.2008.00455.x PMC6512388

