# Supplementary figures and images for: Embryonic Morphogen Nodal Promotes Breast Cancer Growth and Progression
Source: PLoS One. 2012 Nov 7;7(11):e48237. doi: 10.1371/journal.pone.0048237 (PMC3492336; doi:10.1371/journal.pone.0048237)

**A** 231+shControl 4 weeks (Brain)

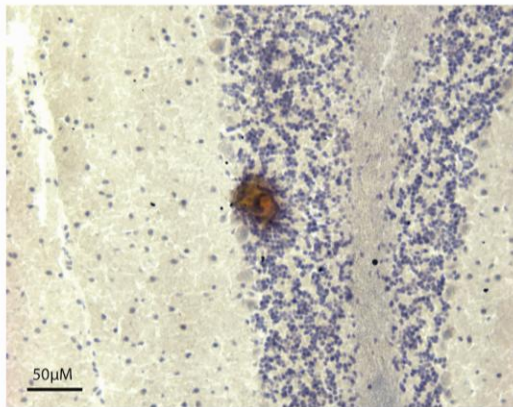

**B** 231+shControl 8 weeks (Liver)

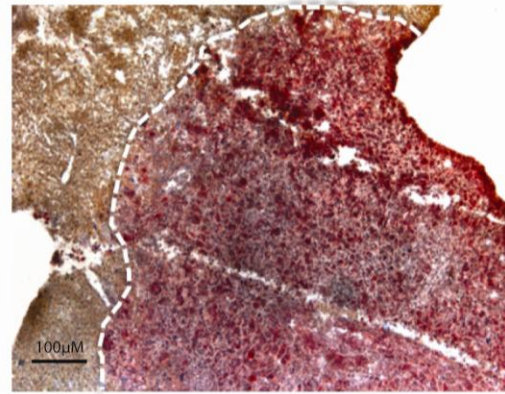

Supplement: Figure S1 — Nodal supports tumour metastasis. (A) GUSB staining of a brain metastasis from MDA-MB-231 cells transfected with a Control shRNA (231+shControl) in NOD/SCID/MPSVII mice 4 weeks post-intravenous injection (red). (B) GUSB staining demonstrates a liver macrometastasis from 231+shControl cells after 8 weeks. No tumours were found in either brain or liver from 231+shNodal cells at either 4 or 8 weeks. (PDF) [file pone.0048237.s001.pdf]
