# Supplementary material for: Embryonic Morphogen Nodal Promotes Breast Cancer Growth and Progression
Source: PLoS One. 2012 Nov 7;7(11):e48237. doi: 10.1371/journal.pone.0048237 (PMC3492336; doi:10.1371/journal.pone.0048237)
Supplement: Table S1 — (PDF) [file pone.0048237.s002.pdf]

Table S1: PrimerProbe information for Real-time PCR.

Target	Probe ID
Nodal	Hs00250630_s1
ALK-4 (ACVR1B)	Hs00244715_m1
ALK-7 (ACVR1C)	Hs00377065_m1
Cripto-1	Hs02339499_g1
BAX	Hs00751844_s1
BCL2	Hs00751844_s1
RPLPO	4333761-0707014
HPRT1	4333768-0904021
