# Supplementary material for: Embryonic Morphogen Nodal Promotes Breast Cancer Growth and Progression
Source: PLoS One. 2012 Nov 7;7(11):e48237. doi: 10.1371/journal.pone.0048237 (PMC3492336; doi:10.1371/journal.pone.0048237)
Supplement: Table S2 — (PDF) [file pone.0048237.s003.pdf]

Table S2: Antibodies for Western Blot (WB), Immunofluorescence (IF) and Immunohistochemical (IHC) Analyses

Antibody	Conc & Use	Company
Monoclonal rabbit anti-P-SMAD2	1:1000 (WB)	Millipore
Rabbit anti-SMAD2/3	1:1000 (WB) 1:100 (IF)	Millipore
Monoclonal mouse anti-B-Actin	1:5000 (WB)	Santa Cruz Biotechnology,
Polyclonal rabbit anti-Caspase-9	1:1000 (WB)	Cell Signaling Technology
Polyclonal rabbit anti-cleaved Caspase-9	1:1000 (WB)	Cell Signaling Technology
Polyclonal rabbit anti-phospho Histone H3 (Thr3)	1:1000 (WB)	Cell Signaling Technology
Polyclonal rabbit anti-phospho Histone H3 (Thr11)	1:1000 (WB)	Cell Signaling Technology
Monoclonal rabbit anti-phospho Histone H3 (Ser10)	1:1000 (WB)	Cell Signaling Technology
Polyclonal rabbit anti-phospho Histone H3 (Ser28)	1:1000 (WB)	Cell Signaling Technology
Monoclonal rabbit anti- Histone H3	1:1000 (WB)	Cell Signaling Technology
Monoclonal rabbit anti-hKi67, clone SP6	Ready-to-use (IHC)	Thermo Scientific
TUNEL	1:100 (IHC)	Promega
